# Pediatric Thyroid Disease: When is Surgery Necessary, and Who Should be Operating on Our Children?

**DOI:** 10.4274/Jcrpe.817

**Published:** 2013-03-01

**Authors:** Christopher Breuer, Charles Tuggle, Daniel Solomon, Julie Ann Sosa

**Affiliations:** 1 Ohio State University College of Medicine, Department of Surgery, Division of Pediatric Surgery, Columbus, OH; 2 Yale University School of Medicine, Department of Surgery, New Haven, CT; 3 Duke University, Department of Surgery, Section of Endocrine Surgery, Durham, NC

**Keywords:** Pediatric thyroidectomy, thyroid surgery, outcomes

## Abstract

Surgical diseases of the thyroid in the pediatric population represent a diverse set of both benign and malignant conditions. Overall, incidence is rare. Benign conditions include Graves’ disease, toxic adenomas, congenital hyperthyroidism, and goiter. Differentiated thyroid cancer (DTC) and medullary thyroid carcinoma (MTC), with its related familial cancer syndromes, are the most common malignancies. Near-total or total thyroidectomy is the appropriate surgery for thyroid cancer, with/out central lymph node dissection. Emerging practice guidelines from professional societies are helpful, although they generally have not addressed surgical management of the pediatric patient. Thyroidectomy in children is associated with a higher rate of complications, such as recurrent laryngeal nerve injury and hypoparathyroidism, as compared to the surgery in adults. Therefore, it is essential that pediatric thyroidectomy be performed by high-volume thyroid surgeons, regardless of specialty. Case volume to support surgical expertise usually must be borrowed from the adult experience, given the relative paucity of pediatric thyroidectomies at an institutional level. These surgeons should work as part of a multidisciplinary team that includes pediatric endocrinologists and anesthesiologists, pediatricians, nuclear medicine physicians, and pathologists to afford children the best clinical outcomes.

**Conflict of interest:**None declared.

## INTRODUCTION

Surgical diseases of the thyroid in the pediatric population represent a diverse set of both benign and malignant conditions. Benign conditions include Graves’ disease, toxic adenomas, congenital hyperthyroidism, and goiter. Malignancy is most commonly differentiated thyroid cancers (DTC), which includes papillary (predominantly) and follicular cancers, and medullary thyroid cancer (MTC), with its related familial cancer syndromes. All surgical diseases of the thyroid are rare in children.

**Benign Thyroid Conditions that May Require Surgical Intervention **

Graves’ disease is an autoimmune condition which causes hyperthyroidism or thyrotoxicosis secondary to the production of autoantibodies to the thyroid-stimulating hormone (TSH) receptor. The incidence of Graves’ disease in the pediatric population is approximately 1 per 100 000 per year. This represents 0.1 per 100 000 in the child and 3 per 100 000 in the adolescent population (1). Children with Graves’ disease should be managed with methimazole, and the dose should be adjusted until the patient is rendered biochemically euthyroid and the symptoms of Graves’ disease are relieved; beta-blockers can be added for the cardiovascular sequelae. The use of propylthiouracil (PTU) for management of Graves’ disease in children is prohibited due to the higher incidence of liver failure, which recently led the U.S. Food and Drug Administration (FDA) to issue a warning against the use of PTU for the treatment of Graves’ disease in children (and adults). Children on methimazole should be monitored for side effects including skin rash, joint and muscle pain, fever, and agranulocytosis. If side effects develop, methimazole should be discontinued immediately. When methimazole is chosen as the first-line treatment for Graves’ disease in children, it should be administered for one to two years and then discontinued, or reduced, in order to assess whether the patient is in remission. For patients who cannot be managed with methimazole or who fail to undergo remission, consideration should be given to definitive treatment with surgery or radioactive iodine.

Numerous studies have reported a failure of anti-thyroid drugs (ATD) to produce a lasting remission and to provide a euthyroid state for children with Graves’ disease. In a 25-year retrospective analysis, Hamburger reported that only 59 of 182 (32%) pediatric and adolescent patients with Graves’ disease initially treated with ATD achieved a euthyroid state and lasting remission. The remaining 68% of patients ultimately progressed to definitive therapy with either radioactive iodine or surgery (2). In another retrospective review of 184 children receiving ATD for Graves’ disease, only 28% were able to achieve remission within two years of starting pharmacologic treatment; 53% of patients eventually went on to receive radioactive iodine or undergo thyroidectomy (3). In younger children, the response rate to medical therapy is less robust. In two analyses of pre-pubescent and pubescent children receiving ATD for Graves’ disease, pre-pubescent children were able to achieve lasting disease remission in only 17% of cases after an average of 3.5 years of therapy, and 33% after an average of six years of therapy (4,5). The overwhelming majority of children with Graves’ disease ultimately require definitive management.

Concerns regarding the safety of radioactive iodine therapy in children stem from several studies. In a population-based analysis of 7 417 patients over 41 years, Franklyn et al (6) noted an overall decrease in the incidence of cancers and cancer-specific mortality in hyperthyroid patients who received radioactive iodine; however, there was a significant increase in TC incidence and deaths attributed to TC. A similar multi-institutional retrospective analysis of 21 000 individuals treated with radioactive iodine observed that while mortality from all cancers was not statistically different from the rate expected after 10 years of follow-up, TC incidence and TC-related mortality were nearly four times higher than expected. However, no TC were identified in patients treated with radioactive iodine before age 20 (7). In another study of 107 patients younger than age 20 years treated with radioactive iodine, not a single thyroid or hematopoietic malignancy was identified, even though many of the patients did not undergo ablative therapy (8).

The risk of radiation exposure, however, is age- and dose-dependent (9). Thus, the risk is greatest for young children with large goiters. Accordingly, radioactive iodine is a better option for older children with smaller glands. 131I therapy should be avoided in very young children (age <5 years). 131I therapy in patients between 5 and 10 years of age is generally acceptable if the calculated 131I administered activity is <10 mCi. 131I therapy in patients older than 10 years is acceptable if the activity is >150 μCi/g of thyroid tissue.

The potential association between the development of parathyroid hyperplasia and hyperparathyroidism after radioactive iodine therapy is another point of controversy. Schneider et al (10) evaluated 4296 patients (mean age 8.5 years, standard deviation 12.8 years) who underwent external beam radiation to the head and neck. This study showed an incidence of hyperparathyroidism approaching 5% at 50 years in a dose-dependent manner, and also showed that patients exposed at a younger age had the highest risk for developing the disease (10). A strong association between radiation exposure and hyperparathyroidism also has been seen in several adult case series (11,12). However, two recent large cohort studies have disputed these results. No increased incidence of hyperparathyroidism was observed in 3 440 individuals who grew up in proximity to the Hanford Nuclear site (13), or in a Swedish cohort of children and adolescents (mean age 7.4 years) treated with radioactive iodine for thyrotoxicosis (14).

or thySurgery is an acceptable form of therapy for pediatric Graves‘ disease. Thyroid surgery should be chosen when definitive therapy for Graves’ disease is indicated, and the child is too young for 131I or the required dose of 131I is too high. The operation of choice is total or near-total thyroidectomy, performed with the intent of rendering the patient hypothyroid. Subtotal thyroidectomy should be avoided due to an unacceptably high incidence of recurrence. In order to avoid the possibility of hemodynamic instability roid storm in the operating room, children with Graves’ disease undergoing thyroidectomy should be euthyroid prior to surgery, and methimazole with/out beta-blockers given as necessary. Methimazole is typically given for one to two months in preparation for thyroidectomy. Potassium iodide should be given in the immediate preoperative period. Ten days prior to surgery, potassium iodide (SSKI; 50 mg iodide/drop) can be given as 3-7 drops (i.e., 0.15 to 0.35 mL) three times daily for 10 days prior to surgery. Beta-blockers also may be needed in patients with persistent tachycardia or allergy to methimazole. 

Graves’ patients who undergo thyroidectomy are more likely to suffer from hypocalcemic complications compared to patients who undergo thyroidectomy for other indications (e.g. cancer) (15). There are multiple hypotheses for this finding, including hungry bone syndrome, increased secretion of calcitonin as a result of thyroid manipulation, and increased vascularity and inflammation of the gland which may cause bleeding that can obscure the operative field or result in direct adhesions to the parathyroid glands. At our institution, calcitriol (25-50 mg twice per day) is started three days preoperatively as prophylaxis for post-operative hypocalcemia.

The most common risks of thyroidectomy include recurrent laryngeal or superior laryngeal nerve injury and hypoparathyroidism, any of which may be temporary or permanent. Other less common risks include bleeding, which can occur up to 72 hours after the time of surgery, and wound infection. The largest published series of thyroidectomies for Graves’ disease in children and adolescents included 78 children from the Mayo Clinic over 17 years (16). In this large, single-institution series from a high-volume endocrine surgery center, 96% of patients achieved a permanently hypothyroid state, and none sustained the potential complications of permanent hypocalcemia or permanent recurrent laryngeal nerve injury. Surgical pathology revealed an occult neoplasm in 6% of the patients. In another series of 15 pediatric patients who underwent thyroidectomy for benign thyroid disease (12 for Graves’), a hypothyroid state was achieved in all patients, with one case (6%) of permanent hypocalcemia, and no recurrent laryngeal nerve injuries (17). These results are similar to those in adult patients treated by high-volume endocrine surgeons (18). In a cross-sectional analysis of the Health Care Cost and Utilization Project Nationwide Inpatient Sample (HCUP-NIS), an endocrine-specific complication rate of 5.9% was shown for children undergoing thyroidectomy for benign disease when the procedure was performed by high-volume thyroid surgeons (19).

Practice guidelines regarding the management of hyperthyroidism and other causes of thyrotoxicosis were issued by the American Thyroid Association (ATA) and the American Association of Clinical Endocrinologists in 2011(20); in these guidelines, there is a section committed to the pediatric patient. In a study by Read et al (8), radioactive iodine produced permanent hypothyroidism in 94% of patients, compared to 96-100% for surgery. The long-term complications of radioactive iodine are unclear, while the major complication of pediatric thyroidectomy for Graves’ disease has been demonstrated to be permanent hypocalcemia, occurring in 0-6% of cases, in addition to the risk of recurrent laryngeal nerve injury and postoperative hemorrhage. The authors recommended total thyroidectomy for young patients (<5 years) and those with large goiters (>80g), and radioactive iodine for teenage patients with normal-sized glands. For other patients, the decision for surgery or radioactive iodine should be made by patients and their families, informed by surgeons, endocrinologists, and pediatricians.

Another benign pediatric thyroid disease that can necessitate surgery is a toxic adenoma, or “hot nodule?. Toxic adenomas are autonomously functioning benign tumors that cause symptomatic hyperthyroidism. The true incidence of toxic adenomas in children is too low for accurate epidemiologic estimates (21). In the case of children with solitary or unilobar toxic adenomas, thyroid lobectomy is the recommended procedure (or rarely, isthmusectomy, if the toxic nodule is in the isthmus) (22). Subtotal lobectomy or nodulectomy are not adequate resections and increase the risk of recurrence. As in the case of Graves’ disease, these patients should be rendered biochemically and clinically euthyroid prior to surgery. The risk of lobectomy includes bleeding and recurrent laryngeal nerve injury. 

Hyperthyroidism can also be due to congenital causes. In 1982, Leclere et al (23) identified four generations of a French family affected by toxic thyroid hyperplasia in the absence of TSH-receptor or thyroperoxidase antibodies and without lymphocytic infiltration on pathology. The authors described the new syndrome as “familial non-autoimmune hyperthyroidism” (23). Subsequently, numerous constitutively active TSH-receptor gene mutations have been identified (24,25). These mutations may exist as germline mutations and present as toxic multinodular goiters, or they may represent de novo heterozygous point mutations contained within solitary toxic adenomas. TSH-receptor mutations are notoriously resistant to treatment with ATDs. Therefore, near-total or total thyroidectomy is the treatment of choice. Non-autoimune hyperthyroidism should be suspected in patients with a known family history, or with persistent neonatal or early childhood hyperthyroidism refractory to medical therapy. Genetic testing is available in these cases (26).

A third benign thyroid disease that can require surgical intervention is congenital goiter. Congenital goiters can be uninodular or multinodular. Once again, the true incidence of these pathologies is low enough to preclude epidemiologic estimates (21). Large goiters can be symptomatic. If a patient develops symptoms of compression related to mass effect, such as discomfort or pain (“globus sensation?), dysphagia, dysphonia, or difficulty in breathing particularly when lying flat, surgery should be considered. Uninodular goiters are amenable to lobectomy, while multinodular goiters require near-total or total thyroidectomy.

**Thyroid Malignancies Uniformly Require Surgical Intervention**

Thyroid nodules occur rarely in children but require investigation because the incidence of malignancy is higher in the pediatric population than in adults (27). Evaluation of a thyroid nodule should begin with a history and physical examination, along with a biochemical evaluation. This is typically followed by an ultrasound. In patients who are hyperthyroid, ultrasound should be followed by a nuclear medicine (“uptake?) scan in order to determine if the pathology is a “warm? or “hot? nodule (that is, a toxic adenoma). Suspicious nodules should undergo interrogation with an ultrasound-guided fine-needle aspiration (FNAB). If the evaluation of a thyroid nodule reveals an FNAB with cytology that is consistent with follicular or Hürthle cell neoplasia, surgical excision is warranted for definitive histologic diagnosis; cytology cannot identify vascular or capsular invasion and discriminate adenomas from carcinomas. There are no specific professional society guidelines addressing the approach to follicular or Hürthle cell neoplasms in children. In these cases, we recommend thyroid lobectomy with completion thyroidectomy if the pathology demonstrates vascular and/or capsular invasion, except in the case of bilateral nodularity, where a near-total or total thyroidectomy is warranted initially. Malignant lesions should be managed as described below.

According to the most recent published data from the Surveillance, Epidemiology and End Results (SEER) database, the age-adjusted annual incidence of thyroid cancer in the United States is 0.54 per 100 000 per year (28). Incidence of DTC is 0.49 per 100 000 per year, but is rising steeply, largely because of the rapid expansion of papillary thyroid cancer that has been observed around the world. Compared to adults, pediatric patients with DTC present with more extensive disease; lymph node involvement at the time of diagnosis is seen in 40-80% of children compared to 20-50% of adults. Children present with distant metastases in 20-30% of cases (29). Nevertheless, prognosis is favorable, with an associated 5-year survival of 95-99%, and a 20-year survival of 90% (30). Owing to the relative rarity of DTC in children, there have been no prospective randomized clinical trials to determine optimal management. 

The Surgical Disciplines Committee of the Children’s Cancer Groups has published two studies advocating for extensive surgical resection at the time of diagnosis. In a retrospective review of 329 patients diagnosed with DTC prior to age 21, the investigators found that progression-free survival was inversely associated with age and directly associated with the presence of residual cervical disease after surgery (31). In a follow-up study of 83 patients from the original cohort who presented with metastatic disease at the time of diagnosis, the investigators noted that the 10-year overall survival was 100%. All of the patients in this subgroup received radioactive iodine in the adjuvant setting (32). In a similar study of 235 patients diagnosed with DTC before age 18, Handkiewicz-Junak et al (33) demonstrated that anything less than a total thyroidectomy was associated with an increased risk of local recurrence in the thyroid bed. When thorough lymphadenectomy was performed in conjunction with near-total or total thyroidectomy, patients with clinically or radiographically positive lymph nodes achieved survival rates that were similar to those of patients who presented with node-negative disease. In this particular case, the authors defined lymph node management as “complete” if it included a modified radical neck dissection (33).

Moreover, PTC is frequently multifocal and bilateral within the thyroid gland; therefore, near-total or total thyroidectomy is usually indicated. This operative approach further facilitates the administration of radioactive iodine post-operatively and long-term surveillance with thyroglobulin (Tgb) levels. Data also show that risk of recurrence and even survival are enhanced statistically with near-total or total thyroidectomy compared to lobectomy in adults (34). Consequently, there is growing consensus that children with DTC should undergo total or near-total thyroidectomy with central compartment lymph node dissection for clinically or radiographically positive lymph node disease (29). 

Notably, there is little regarding the management of DTC in children among current practice guidelines; only the British Thyroid Association among the major international professional societies specifically addresses the management of DTC in the pediatric population with the broad-based recommendation that “the general principles of management are similar to those in adults” (35). 

MTC arises from the calcitonin-producing parafollicular C cells of the thyroid gland. It has an incidence in children of 0.03 per 100 000 population per year. MTC can occur sporadically or as part of a spectrum of familial MTC syndromes. In adults, sporadic MTC accounts for 65% to 75% of MTC (36), but in the pediatric population, sporadic carcinomas are exceedingly rare; the vast majority of MTC diagnosed in childhood is hereditary (37,38). Hereditary MTC can occur independently as familial MTC (FMTC). It can also occur as part of a triad in the multiple endocrine neoplasia (MEN) syndromes with pheochromocytoma and primary hyperparathyroidism in MEN2A, or with pheochromocytoma, marfanoid habitus, and mucosal neuromas with MEN2B. As such, it is nearly always the first component of the MEN2 phenotypes. Both of these syndromes are related to selected mutations of the RET proto-oncogene receptor tyrosine kinase (39). The MTC related to MEN2B is the most virulent, followed by that of MEN2A and FMTC, respectively. Patients (e.g., parents and parents-to-be) with MTC should be informed about the possibility of inheritable cancer syndromes and offered genetic testing and counseling as appropriate (40).

Advances in our understanding of the genotype–phenotype relationship have allowed for the development of guidelines to direct the timing of prophylactic total thyroidectomy, depending on patient-specific RET mutations. In 2009, ATA issued evidence-based guidelines regarding the management of MTC. Mutations are classified based on time to development of MTC and stratified from A to D (Table 1). Risk D mutations require prophylactic thyroidectomy within the first year of life, while the ATA suggests surgery before age five for A, B, and C mutations, with specific clinical criteria for exception (41). In patients with hereditary or sporadic MTC diagnosed clinically, 80% already have metastasized to ipsilateral and 40% to contralateral cervical lymph nodes (42). In these cases, the ATA guidelines suggest total thyroidectomy and central neck dissection, along with lateral compartment lymph node dissection for clinically or radiographically evident lymphadenopthy (41). 

Post-operatively, patients require diligent surveillance for disease recurrence. Biochemical evidence of disease recurrence includes new calcitonin elevation, or rapid calcitonin or carcinoembryonic antigen (CEA) doubling time. Palpable disease in the surgical bed or draining nodes is also indicative of recurrent disease. In cases of suspected recurrence, imaging with ultrasound is indicated, with the diagnosis confirmed by FNA (40). If extra-cervical disease is suspected, computed tomography (and magnetic resonance imaging for the liver, in particular) can facilitate confirmation.

**Who Should be Operating on Our Children?**

Surgeons with varied specialty training, including pediatric surgeons, otolaryngologist-head and neck surgeons, general surgeons, and endocrine surgeons perform thyroidectomy in pediatric patients. Some surgeon characteristics appear to be associated with improved patient outcomes. A multidisciplinary approach that involves pediatric endocrinologists, pediatricians, surgeons, nuclear medicine physicians, anesthesiologists, and pathologists is an essential part of the preoperative, postoperative, and long-term management of children with thyroid disease, and this is especially poignant for those pediatric patients who undergo thyroidectomy. 

As in adults, surgeon volume appears to be directly associated in a dose-response fashion with patient outcomes in children undergoing thyroidectomy (43). In a study using national data with patients and surgeons from all types of healthcare settings, surgeon volume (in adults and children) had a greater impact on patient outcomes than surgeon specialty (44). In-hospital endocrine-related complication rates tended to be lower for high-volume surgeons compared to low-volume surgeons (5.6% vs. 10%; p=NS). Of note, these complication rates are higher than the rates of 1-2% reported for adults undergoing similar procedures (45). High-volume surgeons had significantly shorter length of hospital stay (1.5 vs. 2.1 days; p<0.05) and costs ($12 474 vs. 15 662; p<0.05) than low-volume surgeons. The challenge in pediatric endocrine surgery is that overall experience is limited because pediatric endocrine diseases requiring operative intervention are relatively rare. These findings suggest that surgeons with the best outcomes sustain their experience by operating on children and adults.

In a literature review examining over 20 case series reports on a total of 1800 pediatric patients, rates of permanent recurrent laryngeal nerve injury and permanent hypoparathyroidism were lowest in operations by high-volume thyroid surgeons (46). The reported range of complication rates was considerable: nerve injury rates ranged from 0% to 40%, and hypoparathyroidism rates varied from 0% to 32%. The authors go on to note, “all surgery should be performed by high-volume, experienced endocrine, pediatric, and head and neck surgeons”. In a review article supporting thyroidectomy as the optimal treatment for pediatric Graves’ disease, Lee et al. (47) suggested that patients travel outside their local area if necessary to find surgeons with extensive experience in pediatric thyroidectomy.

Published guidelines, including the ATA Management Guidelines for Patients with Thyroid Nodules and Differentiated Thyroid Cancer and the National Comprehensive Cancer Network (NCCN) Clinical Practice Guidelines in Oncology, are clear in acknowledging the importance of surgeon volume for patient outcomes in adults undergoing thyroidectomy (48,49). Fewer studies have examined the effect of surgeon volume on surgical outcomes specifically among pediatric patients. The ATA Medullary Thyroid Cancer Management Guidelines address the role of surgeon experience in pediatric outcomes, asserting that children undergoing thyroidectomy have higher complication rates than adults, and have better outcomes when operated on by high-volume surgeons (41). 

The adoption of these recommendations has been evidenced by recent reports describing the experience of hospitals with significant experience in pediatric thyroidectomy. These reports highlight the benefits of increased volume at both the surgeon and institutional level (50,51). High-volume centers are more likely to have systematic approaches to the pre-operative workup and post-operative care of these patients, as well as early involvement of dedicated pediatric and endocrine specialists. Recently, experience with these multidisciplinary, team-based approaches has been described; however, an improvement in outcomes has yet to be proven (52).

Surgeon volume appears to be the most important and robust predictor of pediatric outcomes after thyroidectomy, and it appears to be independent of the effect of surgeon specialty. Ensuring access to high-volume surgeons requires a multidisciplinary approach involving parents, pediatricians, and pediatric endocrinologists (53). For example, high-volume surgeons need to work with pediatricians in the management of children with postoperative hypocalcemia, which is not uncommon after thyroidectomy. The effects of postoperative complications in children are particularly poignant, as they can have profound effects on physical and psychosocial development as well as quality of life for decades afterward. Collaboration between high-volume pediatric surgeons and (adult) thyroid surgeons, parents, and pediatricians is likely to optimize patient outcomes.

## Figures and Tables

**Table 1:**
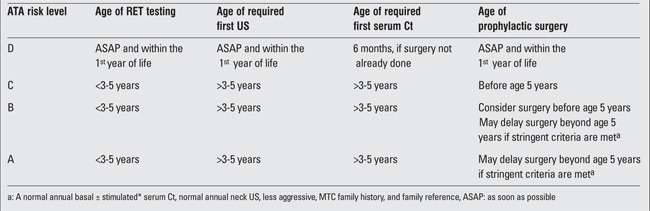
American Thyroid Association (ATA) risk level and prophylactic thyroidectomy testing and therapy
